# Code of Dental Ethics

**Published:** 1866-08

**Authors:** 


### CODE OF DENTAL ETHICS.

(Adopted by the Ohio State Dental Association.)

### ARTICLE I.

*Duties of Dentists to their Patients.*

1. As far as possible the dentist should be always ready to respond to the wants of those requiring his services. He should fully recognize the responsibility resting upon him in the discharge of his duty. Faithfulness in the performance of his operations depends chiefly on his own sense of right, rather than the patient's ability to judge of their character. He should bear in mind that the comfort, health, and, to some extent, the lives of his patients are committed to his charge, and depend upon his skill and faithfulness. He should manifest a kind and sympathizing disposition, combined with firmness in doing that which is right. He should exercise proper authority, as far as his knowledge and judgment will warrant. His whole deportment should be such as to secure respect and confidence.

2. He should treat every case committed to his care with attention, humanity and firmness, remembering that most of his operations are performed on living tissues, and may produce great suffering. He should make due allowonce for the want of accurate knowledge on the part of his patient of many things which seem so clear to himself. He should always exhibit to his patient a correct deportment, in all his bearings, and in nothing more than in his statements to them in regard to professional matters. These should be not only true, but clear, and calculated neither to magnify nor to diminish—to represent a case neither as more nor less difficult than it is. He should not promise certainty when, in the nature of the case, there is uncertainty. His deeds, rather than his tongue should declare his ability.

3. To insure to his patients the best results, the dentist should aim to secure, and to preserve in himself, both physical and mental health, avoiding influences calculated to unfit him for achieving the highest attainments. He ever needs a clear head, a pure heart, and strong hands.

### ARTICLE II.

#### *Maintaining Professional Character.*

1. When a man enters the dental profession, as he becomes invested with its privileges, so he incurs all its obligations. He is bound to maintain its honor and to labor earnestly to extend the sphere of its usefulness. He should avoid everything in language and conduct, calculated to discredit or dishonor the profession, and should show all due respect to his brethren, especially to his seniors.

2. The dentist owes, both to his profession and his patients, the duty of maintaining a high-toned moral character. He should also cultivate genteel manners. Any lack of neatness and cleanliness, either in person or in office arrangements, is disrespectful to his brethren, as well as to his patients.

3. It is unprofessional to resort to public advertisements, cards, handbills, or posters, calling attention to peculiar styles of work, lowness of prices, special modes of operating, or claiming superiority over neighboring practitioners, publishing reports of cases, or certificates, in the public prints, going from house to house to solicit or perform operations, circulating or recommending nostrums, or performing any other similar acts.

4. When a dentist is consulted by a patient of another practitioner, he should practice due caution in regard to inquiries or hints disparaging to the family dentist, or calculated to weaken the patient's confidence in him; and, if the best interests of the patient will not be sacrificed thereby, the case should be temporarily treated, and referred back to the family dentist.

5. When practicable, some general rules should be adopted, by members of the profession practicing in the same localities, in relation to fees; and it is dishonorable and unprofessional to depart from these rules except when variation of circumstances requires it. And it is ever to be regarded as unprofessional to warrant operations or work, as an inducement to patronage.

### ARTICLE III.

*The Relative Duties of Dentists and Physicians.*

Dental Surgery is a speciality in medical science. Members of both professions should bear this in mind. The dentist is professionally limited to diseases of the dental organs and the mouth. With these he should be more familiar than the general practitioner is expected to be; and, while he recognizes the superiority of the physician, in regard to diseases of the general system, the latter is under equal obligation to respect his higher claims, in reference to his specialty. Where this principal governs, there can be no conflict, or even diversity of professional interests.

### ARTICLE IV.

*The Mutual Duties of the Profession and the Public.*

Dentists are frequent witnesses, and, at the same time, the best judges of the impositions perpetrated by quacks; and it is their duty to enlighten and warn the public in regard to them. For this, and for the other benefits conferred by the competent and honorable dentists, the profession is entitled to the confidence and respect of the public, who should ever discriminate in favor of the true man of science and integrity, and against the empiric and impostor.

